# Development of genomic resources for *Citrus clementina*: Characterization of three deep-coverage BAC libraries and analysis of 46,000 BAC end sequences

**DOI:** 10.1186/1471-2164-9-423

**Published:** 2008-09-18

**Authors:** Javier Terol, M Angel Naranjo, Patrick Ollitrault, Manuel Talon

**Affiliations:** 1 Centro de Genómica, Instituto Valenciano de Investigaciones Agrarias, Carretera Moncada, Náquera, Km. 4.5 Moncada, Valencia, E46113, Spain; 2 CIRAD, UPR 75, Avenue Agropolis, TA A-75/02, 34398 Montpellier, Cedex 5, France

## Abstract

**Background:**

Citrus species constitute one of the major tree fruit crops of the subtropical regions with great economic importance. However, their peculiar reproductive characteristics, low genetic diversity and the long-term nature of tree breeding mostly impair citrus variety improvement. In woody plants, genomic science holds promise of improvements and in the *Citrus *genera the development of genomic tools may be crucial for further crop improvements. In this work we report the characterization of three BAC libraries from Clementine (*Citrus clementina*), one of the most relevant citrus fresh fruit market cultivars, and the analyses of 46.000 BAC end sequences. Clementine is a diploid plant with an estimated haploid genome size of 367 Mb and 2n = 18 chromosomes, which makes feasible the use of genomics tools to boost genetic improvement.

**Results:**

Three genomic BAC libraries of *Citrus clementina *were constructed through *Eco*RI, *Mbo*I and *Hind*III digestions and 56,000 clones, representing an estimated genomic coverage of 19.5 haploid genome-equivalents, were picked. BAC end sequencing (BES) of 28,000 clones produced 28.1 Mb of genomic sequence that allowed the identification of the repetitive fraction (12.5% of the genome) and estimation of gene content (31,000 genes) of this species. BES analyses identified 3,800 SSRs and 6,617 putative SNPs. Comparative genomic studies showed that citrus gene homology and microsyntheny with *Populus trichocarpa *was rather higher than with *Arabidopsis thaliana*, a species phylogenetically closer to citrus.

**Conclusion:**

In this work, we report the characterization of three BAC libraries from *C. clementina*, and a new set of genomic resources that may be useful for isolation of genes underlying economically important traits, physical mapping and eventually crop improvement in *Citrus *species. In addition, BAC end sequencing has provided a first insight on the basic structure and organization of the citrus genome and has yielded valuable molecular markers for genetic mapping and cloning of genes of agricultural interest. Paired end sequences also may be very helpful for whole-genome sequencing programs.

## Background

Citrus, one of the major fruit tree crops is widely cultivated throughout the globe and therefore has a tremendous economical, social and cultural impact in our society. Citrus improvement through traditional techniques, however, is highly impaired due to the unusual combination of biological characteristics of *Citrus *species, their low genetic diversity and the long-term nature of tree breeding. Citrus are diploid plants with an estimated haploid genome size of about 367 Mb and 2n = 18 chromosomes, which may facilitate the use of genomics tools for crop improvement. Expressed sequence tag (EST) analyses and molecular marker studies strongly suggest that the main commercial citrus cultivars (oranges, lemons and grapefruits) are mostly interspecific hybrids and therefore are heterozygous "species" [1]. In addition, most of the cultivars in these groups, including Clementine varieties, may represent accumulated somatic mutations identified over centuries [2].

The development of citrus genomic resources is in its infancy although in recent years major efforts and goals mostly on functional genomics have certainly been undertaken [3]. Critical functional and expression analyses through microarrays with several platforms have also been published and analyses of ESTs in public databases have been initiated [4,5]. For instance, 401,692 citrus ESTs have been deposited at GenBank and are currently available. This collection constitutes a valuable source for the direct access to the genes of interest and for the development of molecular markers for map-based cloning purposes or marker-assisted selection programs [6-9]. Moreover, genetic linkage maps have been produced with increasing value and resolution, following the evolution of new marker systems [10,11]. Genetic transformation in citrus is also available [12] and strategies based on genome-wide mutagenesis are being explored. Other innovative resources such as viral-induced gene silencing (VIGS) are being developed and work in citrus proteomics is in progress [13,14]. Thus, current advances in citrus research include the rapid development of functional genomics and molecular biology resources [15] although, on the other hand, basic information on the organization and structure of citrus genome is lacking. The main challenge for a comprehensive and meaningful description of genomes is the integration of the DNA marker-based genetic maps with physical maps, and eventually with DNA sequence of the whole genome, the ultimate physical map. For the generation of high-resolution physical maps, the construction of BAC libraries containing clones with large DNA fragments appears to be indispensable. BAC end sequencing is indeed an important component of physical map development and can be considered a form of low coverage sequencing [16]. Paired end sequences of BACs form an important part of scaffolding whole-genome shotgun programs [17] as well as in BAC based genome projects [18,19]. In addition, BAC clone collections and BAC-based contig maps are powerful tools having multiple applications in genomics including positional cloning. The BAC end sequence provides a random survey of the information contents (genes, transposons, repeats) of unsequenced genomes [20-22], and yields molecular markers useful for genetic mapping [23-25], and cloning of genes of agricultural interest [26-28]. Furthermore, in many agriculturally important species BAC clones and physical maps are being rapidly developed since they are essential components in linking phenotypic traits to the responsible genetic variation, to integrate the genetic data, for the comparative analysis of genomes, and to speed up marker-assisted selection (MAS) for breeding. Thus, BAC libraries have become central for physical mapping, genome analysis, clone based sequencing and sequencing of complex genomes, for both model [18,19] and main crop plants [29-32].

In citrus, two BAC libraries from *Poncirus trifoliata *[11] and a hybrid of *Citrus *× *Poncirus *[10] have been described in detail. These libraries were constructed as part of a map-based cloning strategy of genes conferring resistance to citrus tristeza virus that causes significant economic damage and losses to citrus worldwide. *Poncirus *is a non domesticated genus related to citrus species that produces inedible fruit. However, other efforts to generate BAC citrus resources, for instance in Satsuma or sweet orange have also been accomplished [3].

In this work, we report the characterization of three genomic BAC libraries from Clementine (*Citrus clementina*) mandarin, a cultivar of great [3]economic importance that has been a main target of recent studies [15]. The development of these new genomic tools also complements the functional genomics platform generated for this species including an extensive EST collection and a 20 k cDNA chip [4,5], expanding further possibilities for isolation of genes of agronomical interest [33,34]. This study provides the most comprehensive, large insert clone resource of any *Citrus *species and reports the analysis of 46,339 BAC end sequences offering a first detailed insight into the sequence composition of the Clementine genome. The analyses focused on protein coding regions, repeat element composition, microsatellite and single nucleotide polymorphism contents. Additionally, data on gene homology based comparative genomics with poplar and *Arabidopsis *are also presented. The annotated BAC-end sequences may well serve as useful resources for physical mapping, positional cloning, genetic marker development and genome sequencing of *C. clementina*.

## Results and discussion

### BAC library characterization

Three genomic BAC libraries (CCL1, CCER1 and CCH3) were constructed as described in Material and Methods, with DNA from *Citrus clementina *(var. Clemenules), pEBAC1 as the cloning vector and three different restriction enzymes (Table 1). The CCL1 library composed of 19,200 clones was generated with *Mbo*I partial digestions. *Eco*RI and *Hind*III were used for the construction of CCER1 and CCH3, respectively, and 18,432 clones were picked from each one. The three libraries contained 56,064 BAC clones that were arrayed in 146 384-well microtiter plates. It has been reported that the use of three different restriction enzymes resulted in a more accurate coverage of the genomes, since the different GC contents of their recognition sites increases the representation of a higher number of genomic regions [35]. A single library constructed with one restriction enzyme usually cannot provide a full coverage of the genome, as the restriction sites are not uniformly distributed along the genome, and therefore genomic regions having too many or too few restriction sites are not equally represented [36]. Generally, two or more complementary large insert libraries, constructed with different restriction enzymes, have been successfully used for physical mapping of several plant genomes including those of Arabidopsis[37], japonica rice [38], or soybean [30].

**Table 1:** Genomic *Citrus clementina *BAC Libraries

Library	CCL1	CCER1	CCH3
Vector	pECBAC1	pECBAC1	pECBAC1
Partial digest enzyme	MboI	EcoRI	HindIII
Cloning site	BamHI	EcoRI	HindIII
Average insert size	124 kb	127 kb	132 kb
N° of clones	19,200	18,432	18,432
Missed wells^a^	0.57%	0.37%	0.09%
Blue colonies^b^	0.78%	1.46%	0.19%

^a ^No bacterial growth detected

^b ^Presence of vector but no insert

**Figure 1:**
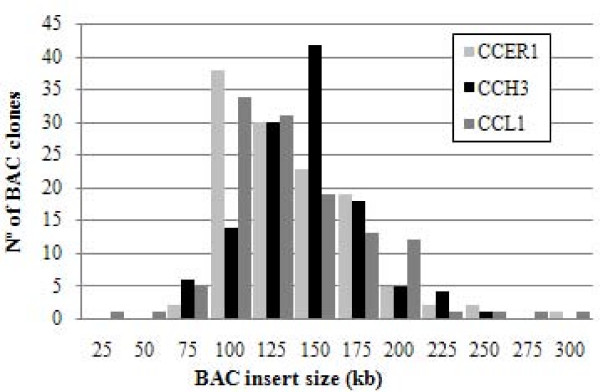
**Size estimate of BAC clones from the CCER1, CCH3 and CCL1 *C. clementina *libraries**. Bars represent the number of BAC clones in each class. 362 BAC clones were randomly selected from the BAC libraries of *C. clementina*: CCER1 (light gray), CCH3 (black), and CCL1 (dark gray).

In order to evaluate the average BAC insert size, 362 BAC clones (about 120 clones from each library) were randomly chosen and the corresponding DNA was extracted, digested with the rare cutter *Not*I enzyme and analyzed by PFGE. All fragments generated by NotI digestion contained the 8.7 kb vector band and various insert fragments (Figure 1). The estimated insert sizes ranged from 10 to 330 kb, with an average of 124 kb for CCL1, 132 kb for CCH3 and 127 kb for CCER1. Since the haploid genome size of *C. clementina *is in the order of 367 Mb, the libraries coverage is predicted to be 19.5 haploid genome equivalents while the probability of finding any specific sequence is greater than 99.999%. It has been estimated that the number of clones representing 10× haploid genomes is adequate for most genome research purposes, including physical mapping [39].

### BAC end sequencing

A total of 28,032 BAC clones from the three genomic libraries were selected for end sequencing as described in material and methods and out of this number, 24,221 clones (86% success rate) rendered 46,339 BAC end sequences (BESs). The three libraries contributed approximately with similar number of reads. The average read length was 652.8 bp and the genomic raw sequence produced was 28.6 Mb, which corresponds to almost 8% of the Clementine genome. Table 2 shows a summary of the BAC end sequencing features. The 46,339 BESs were deposited at GenBank with accession numbers from ET068227 to ET114565.

**Table 2:** *Citrus clementina *BAC end sequencing summary

Library	CCL1	CCER1	CCH3	Total
Processed BACs	9,216	9,216	9,600	28,032
Positive BACs	8581	7262	8378	24,221
Success rate	93.1%	78.8%	87.3%	86.4%
BES mate pairs^a^	8036	6401	7681	22,118
Genomic equivalents^b^	3.1	3.1	3.9	10.1
				
Reads	16,617	13,663	16,059	46,339
Success rate	90%	74%	84%	83%
Total raw sequence (bp)	10,102,329	8,783,997	9,676,620	28,562,946
Average read length (bp)	638.0	679.6	644.3	652.8
Chloro/mito reads^c^	432	146	204	782
Chloro/mito total sequence (bp)	273,452	94,570	111,324	479,346
Final genomic sequence (bp)^d^	9,828,877	8,689,427	9,565,296	28,083,600

^a ^BAC clones with 5' and 3' good quality reads

^b ^Genomic equivalents calculated considering the number of clones sequenced and their average length

^c ^Reads identified as chloroplastic or mitochondrial DNA

^d ^Total genomic sequence obtained, without mitochondrial or chloroplastic DNA

### Sequence Annotation

#### Chloroplast and mitochondrion DNA analysis

In order to identify extranuclear sequences, BES were first compared against the *Citrus sinensis *chloroplastic [40] and the *Arabidopsis thaliana *[41] mitochondrial genome sequences with an stringent threshold of 1e-15. The comparison indicated that 736 (1.68%) and 46 (0.1%) sequences produced significant matches with the chloroplastic and mitochondrial genomes, respectively. Chloroplastic BESs were assembled and the consensus sequences obtained spanned 101,470 bp, approximately 70% of the chloroplast genome. Chloroplastic and mitochondrial DNA summed up to 480 kb, and therefore, the total genomic DNA obtained was 28,1 Mb. Considering the average size of the BAC clones, the coverage provided by the BAC end sequencing was higher than 8.4 genomic equivalents.

#### Repetitive DNA Analysis

**Figure 2:**
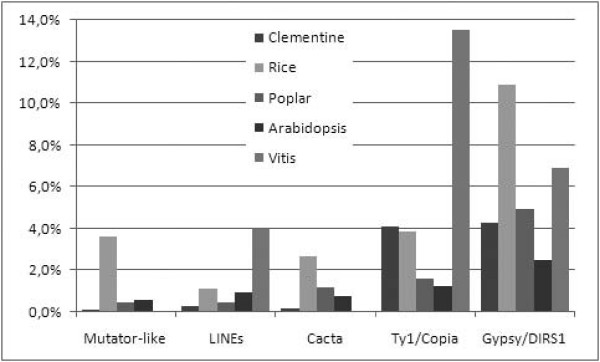
**Comparative analysis of the most abundant transposable elements from *C. clementina***. Estimates of the amount of specific classes of transposable elements are represented as percentage of occupied sequence in *C. clementina*. For comparative analysis, data from *O. sativa *[19], *P. trichocarpa *[17], and *A. thaliana *[18] are included.

The repetitive DNA fraction present in Clementine BESs revealed with RepeatMasker, included 9,618 interspersed repeats which extended over 2.55 Mb, 8.95% of the total raw sequence (Table 3). BLASTX search performed to identify coding regions (see next section), showed that 2,173 additional sequences not detected by RepeatMasker also presented high significant similarity to transposable elements (TEs) and, therefore, are also part of the repetitive DNA fraction of the genome. Thus, the number of BESs with interspersed repeats rose to 11,791, approximately a 25% of the total reads, a percentage between those found for *Carica papaya *(16%) [20] or *Musa acuminata *(36%) [21]. No significant differences between the 3 BAC libraries were found when the number of BESs carrying repetitive elements was compared (see Additional File 1). The sequence length occupied by transposon elements (TEs) including the additional reads identified through homology search was in this way increased to 3.58 Mb, a fraction corresponding to 12.6% of the total BAC end sequences. This fraction was rather similar to the ratio reported for *Brassica rapa *(13.8%) in an estimation also based on partial sequencing [22]. Comparisons with the percentages found in fully sequenced genomes showed that the relative occurrence of TEs in Clementine was also similar to the fractions found in Arabidopsis (10%) [18] and black cottonwood (12.6%) [17] and lower than in rice (35%) [19] and grapevine (38.8%) [42]. Although the proportion of the different TEs largely varied, in all these species, as well as in *C. clementina*, class I elements (including LINE, Gypsy-like and Copia-like elements) were predominant over class II (including CACTA, MULE and hAT elements). In comparison with the above sequenced plants, the occurrence of Mutator-like, LINEs, Cacta, and Gypsy elements, represented as the percentage of occupied sequence, was in general lower in the genome of *C. clementina*. In contrast, copia-like elements were relatively more abundant in Clementine than in *Arabidopsis*, rice and poplar (Figure 2). The abundance of these elements in citrus has previously been estimated to be relatively high (13%) [43], while the data obtained through partial sequencing in this study suggested a lower preponderance (3.9%; Table III). Copia and gypsy like elements, however, are transcriptionally active in Clementine [43,44] and therefore, could be an important source of genetic variability in this species.

**Table 3:** Repetitive DNA in *Citrus clementina *BESs identified by Repeat Masker

	Number of elements	Length (bp)	Sequence (%)	Number of elements (%)
**Retroelements**				
LINEs				
L1/CIN4	360	67,719	0.237%	3.74%
LTR elements				
Ty1/Copia	3,770	1,142,096	3.999%	39.20%
Gypsy/DIRS1	3,917	1,172,005	4.103%	40.73%
Other	265	6,939	0.024%	2.76%
Total Retroelements	8,312	2,388,759	8.363%	86.42%
**DNA transposons**				
hobo-Activator	346	64,065	0.224%	3.60%
Tc1-IS630-Pogo	97	10,162	0.036%	1.01%
En-Spm	381	55,814	0.195%	3.96%
MuDR-IS905	386	29,983	0.105%	4.01%
Tourist/Harbinger	13	1,400	0.005%	0.14%
Other	83	5,653	0.020%	0.86%
Total DNA transposons	1,306	167,077	0.585%	13.58%

**Total interspersed repeats**	9,618	2,555,836	8.948%	

**Figure 3:**
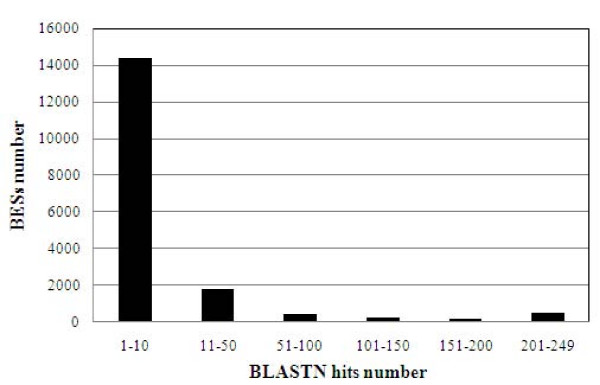
**Putative low complexity repetitive sequences identified through BLASTN search of BESs against themselves**. The figure represents the frequency distribution of BESs as related to the hit number obtained after BLASTN search of BESs against themselves. BESs producing more than 50 copies were considered to be putative interspersed repeats.

In order to identify low complexity repeats, all BESs were searched against themselves with BLASTN and then classified based on the number of significant hits produced (Figure 3). After filtration, 17,585 BES producing at least one hit different from themselves were clustered in this way. The results showed that while a high proportion of BESs (82%) displayed a low number of hits, 3,221 reads produced more that 10 hits, suggesting that these BESs may carry non-coding repetitive sequences, i.e. they may be interspersed repeats of lower complexity. The amount of sequence occupied by these repeats was estimated to be 1.12 Mb, 3.94% of the analyzed sequence. This proportion appears to be moderate in comparison with the 23.5% figure reported for poplar [17].

#### Analysis of coding regions

After filtration of mitochondrial, chloroplastic and repetitive sequences, the remaining 30,787 BES were analyzed for coding region identification via homology search. Parallel searches with BLASTX and BLASTN were performed against the non-redundant database (e value cut off of 1e-4) and a database of *Citrus *ESTs from GenBank (e value cut off of 1e-15), respectively. The BLASTX search identified 14,030 BES (36% of the total BESs) with significant protein hits, while the BLASTN search revealed a similar number of reads, i.e. 14,023 reads that rendered significant homology with 40,536 citrus ESTs. Overall, 20,185 BESs produced BLASTX and/or BLASN hits, and the 3 BAC libraries rendered a similar number of clones carrying potential coding regions (see Additional File 1). The total number of BAC clones that produced protein and/or EST hits was 15.658, while 4,527 of them provided hits in both 5' and 3' ends, an observation that suggested high gene contents in these BAC clones and indeed their possible location at the euchromatin. It was also found that 7,868 BES produced hits for both proteins and ESTs, strongly indicating that they contained active transcription units.

Assuming that each significant BLAST hit corresponds to a different transcription unit, the total number of hypothetical genes described in the BAC ends was 20,185. Thus, considering the amount of analyzed sequence (28.1 Mb), the estimated genome size (367 Mb), and the number of genomic equivalents analyzed (8.4), the gene contents of the genome of *Citrus clementina *was assessed as 31,000. This gene number is comparable to the estimate reported for three species of similar genome size that have been sequenced to completion to date: rice (*Oryza sativa*), with a genome size of 430 Mb and 41,042 genes identified [45], Black cottonwood with 55,000 genes in 485 Mb [17], and grapevine (*Vitis vinifera*), with 30,434 genes in 487 Mb [42]. Other estimates based on BES analysis obtained for plant species were also analogous. For instance, in papaya, the estimated gene contents was 35,526 with a 372 Mb genome size [20], and in Chinese cabbage, with a genome of 529 Mb, 43,000 genes were calculated [22].

**Figure 4:**
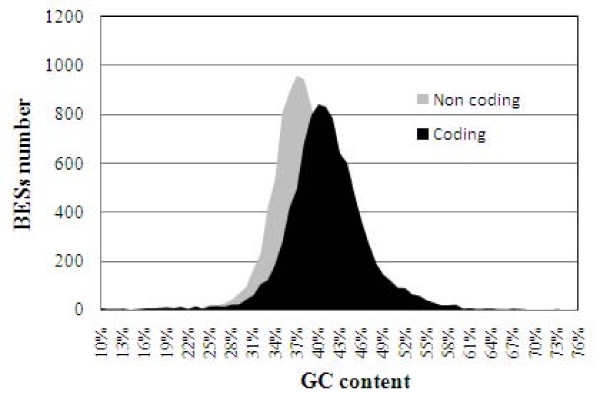
**GC content in BES of *Citrus clementina *BAC libraries**. Distribution of GC content in coding and non-coding regions of BES of *Citrus clementina *BAC libraries.

Furthermore, total GC content in BESs estimated with EMBOSS was 39% while in coding and non-coding sequences, was 41% and 37%, respectively (Figure 4). No significant differences were found when the GC content was compared between the 3 BAC libraries constructed (see Additional File 1). The GC contents reported in other woody plants such as Scots Pine (*Pinus sylvestris*; 39.5%) [46] or yellow-poplar (*Liriodendron tulipifera*; 41%) [28] as well as in coding sequences from Solanaceae species i.e. *Nicotiana tabacum *(40.4%), *Solanum tuberosum *(39.0%), and *Solanum esculentum *(39.8%), or the Fabaceae *Pisum sativum *(39.2%) were also on the same range. The percentage of GC in *Glycine max *(46.5%) and *A. thaliana *(45.4%) was significantly higher [47].

Lastly, Blast2GO [48] was used to analyze the different functions associated with the putative coding regions, and GO terms were assigned to 10,598 sequences. Additional file 2 shows the results obtained for the gene ontology categories Molecular Function and Process. This classification may be useful to identify and locate genes of agronomic interest, such as those related to sugar and cellulose synthesis [GenBank:ET090832, GenBank:ET070671, GenBank:ET074358], ion transport [GenBank:ET110643, GenBank:ET110643, GenBank:ET074792], or calcium metabolism [GenBank:ET068865, GenBank:ET091143, GenBank:ET084632], for instance.

#### SSR Analysis

BAC end sequences have proved to be excellent sources to identify simple sequence repeats (SSRs or microsatellites) in many plant species such as cotton or soybean [23,25,49]. In this work, Sputnik [50] was used to identify a total of 3,814 SSRs longer than 15 bp in the BESs that did not carry repetitive sequences. The occurrence of SSRs in the Clementine genome had a frequency of 0.20 SSR per kb, a value almost identical to that reported in a study based on citrus ESTs (0.19 SSR per kb) [51] and in *B. rapa *(0.18 SSR per kb) [22]. This frequency, however, was lower than the ratios found in grapevine (0.48 SSR per kb) [42], papaya (0.43 SSRs longer than 12 bp per kb) [20], and *A. thaliana *(0.33 SSR per kb).

**Figure 5:**
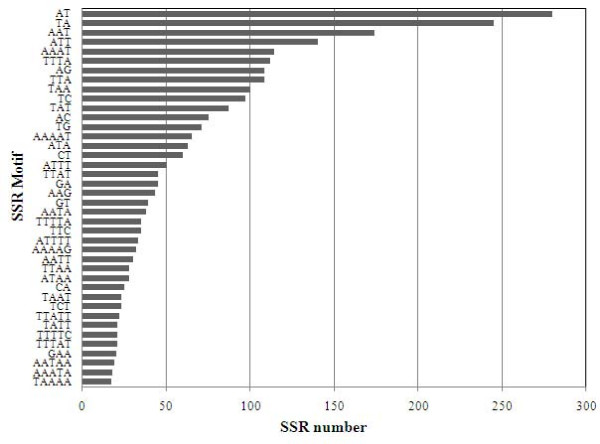
**Number of repeats of the most abundant SSRs in BES of *Citrus clementina *BAC libraries**. Black bars represent the number of repeats found for each SSR motif.

In the Clementine SSR set, there were 758 class I (more than 10 repeats) and 3,056 class II (less than 10 repeats) SSRs, with di and trinucloetides accounting for almost 70% of the SSRs, while tetra and pentanucleotides were less represented. In general, those motifs containing A/T nucleotides were far more abundant than G/C rich repeats, specially ATT/TAA and AT/TA tri- and dinucleotides (Figure 5). A similar distribution was found by Jiang et al[8] and Chen et al. [51] in the analysis of 8,218 and 3,278 citrus SSRs derived from ESTs, respectively. In the Clementine genome, microsatellites were more numerous in non coding sequences (56% of the SSRs) than in putative coding regions (44%), as previously reported in papaya [20], Chinese cabbage [22], and Arabidopsis[18].

Microsatellites are co-dominant, highly polymorphic, and simple to use markers that have been successfully used in studies of genetic diversity [7] and genetic mapping in citrus [8,52] and in many other plants [25,53-55]. The additional markers reported here will certainly contribute to improve the coverage of the *Citrus *genome for many purposes, including the development of accurate linkage and genetic maps.

#### Contig Assembly and SNP analysis

Assembly of BESs that did not contain repetitive sequences was performed with CAP3 [56], and a total of 6,461 contigs including 19,057 reads and covering 6.14 Mb of sequence were produced. It has been suggested that *C. clementina *is an offspring of a *C. sinensis *× *C. reticulata *cross and therefore has a heterozygous genome [1]. In order to identify possible polymorphisms affecting the assembly of the readings and the construction of the physical map of this species, a BLASTN search of all the contigs and singlets was performed against themselves. One hundred thirty sequence pairs that presented a single reciprocal BLASTN hit and the same protein hit from the previous BLASTX search (identical accession number) were selected for further analysis. A total of 81 pairs displayed sequence identities higher than 90% and were considered as originated from the same genomic region. These sequences were not assembled in the same contig due to the presence of polymorphisms, similarly to what was reported in the sequencing of the highly heterozygous grapevine variety, Pinot Noir [57].

The contigs generated in the assembly were analyzed in order to identify single nucleotide polymorphisms (SNPs) in the diploid genome of *C. Clementina*. SNPs are the most abundant and powerful polymorphic markers, since they provide gene-based markers that can be used in the creation of dense genetic linkage maps [58] and, more important, in the identification of genes associated with specific trait loci. BAC end sequences of heterozygous genotypes have also been successfully utilized in SNP discovery and construction of linkage maps [59-61]. PolyBayes [62], a software designed to use genomic sequence as a template and base quality values to discern true allelic variations from sequencing errors, was used to reveal SNP polymorphisms. The number of putative SNPs identified in the Clementine sequences that showed P ≥ 0.9 and SNP depth lower than 10 was 6,617, corresponding to 1.08 SNPs per kb. PolyBayes has been successfully utilized in automated high throughput identification of SNPs from EST collections, and it has been shown that when using P ≥ 0.95, 85% of the predicted SNPs are generally validated experimentally [63].

In order to test the accuracy of the SNP prediction carried out, a total of 30 polymorphisms were randomly chosen for experimental validation. Primers were designed on the consensus sequence of the contigs, in order to amplify the region containing the SNP. Out of 30 genomic regions, 3 produced two or more bands in the PCR amplification or yielded sequence reads with a mixture of templates. This observation constitutes a first indication of the level of heterozygosity of the Clementine genome. Furthermore, the sequence analysis of the remaining SNP candidates showed that 24 out of the 27 hypothetical polymorphisms, an 88.9% success rate, were certainly validated (see Additional File 3). Considering the reliability of the prediction method, more than 5000 putative polymorphisms with P ≥ 0.95 were found in this work. Transitions were the most abundant changes (3,546; 53.6%), followed by transversions (2,162; 32.7%) and indels (909; 13.7%) (Table 4). The transition fraction found in poplar (70%) was substantially higher [17]. The predominant transversion was A/T at a frequency that doubled that of C/G changes (Table 4), an unexpected observation that remains to be explained.

**Table 4:** SNPs in *Citrus clementina *BESs identified by PolyBayes

Summary	
N° SNPs^a^	6617
P_SNP^b^	0.984
Total seq (kb)^c^	6139
SNP/kb	1.08

Detailed relation of SNPs	

Transitions	3546 (53.59%)
ag	1781
ct	1765
Transversions	2162 (32.67%)
ac	541
at	713
cg	330
gt	578
Indels	909 (13.74%)
c-	112
t-	354
g-	108
a-	335

^a ^Number of SNPs with P_SNP > 0,9

^b ^Average PolyBayes score

^c ^Total length of analyzed sequence

It should be noted that the SNP listing reported in this work constitutes the first set of putative SNPs identified in any *Citrus *species, and hence provides a completely new resource for genome analysis in this genera. It is also worth mentioning that 4,500 SNPs were located in or close to putative coding regions, and therefore these 'functional SNPs' may provide an inestimable resource for the identification of genes associated with specific trait loci in addition to their utility as molecular markers for genetic and comparative mapping, nucleotide diversity analysis and association studies.

Two heterozygous genomes have been sequenced to completion, the Nisqually-1 poplar and the Pinot Noir grapevine strains. In both cases the frequency of polymorphisms found within these heterozygous genomes, was 2.6 [17] and 4 [57] SNPs per kb, respectively. These rates are in contrast with the frequency of 1.08 SNPs per kb found in Clementine. The reproductive biology of the different species could explain these differences. Gametophytic self- and cross-incompatibility, and apomixy would produce low variability within *Citrus *species [7], while outcrossing by means of insect and wind pollination, which is the norm for poplar and vitis, would result in highly heterozygous cultivars [17,57].

### Comparative Genomics

The non-repetitive fraction of the BESs was also used in a BLASTN search against the complete nucleotide sequence of the genomes of *A. thaliana*, *P. trichocarpa *and *O. sativa*, with 1e-14 as cut off value. The genomic sequences were displayed with chromosomes as single searchable fasta sequences. In order to map the BESs unambiguously on the heterologous complete genomes, only those sequences producing single significant hits were taken into account. Table 5 shows that the *Populus *genome not only yielded the largest number of significant hits (3-fold more than *Arabidopsis *and almost 5-fold than rice), but also spanned more than twice the length of the sequence displaying similarity.

**Table 5:** Genomic BLASTN results with non-repetitive *Citrus clementina *BESs

	Rice	Arabidopsis	Poplar
Hits	324	443	1,567
Total length (kb)^a^	177.9	235.7	847.5
Average e value	1.8E-09	1.4E-06	2.1E-07
Average distance between hits (kb)	1,545.4	264.3	213.5
Average n° of hits per chromosome	27	88.6	82.5

^a ^Total length of the aligned regions

**Figure 6:**
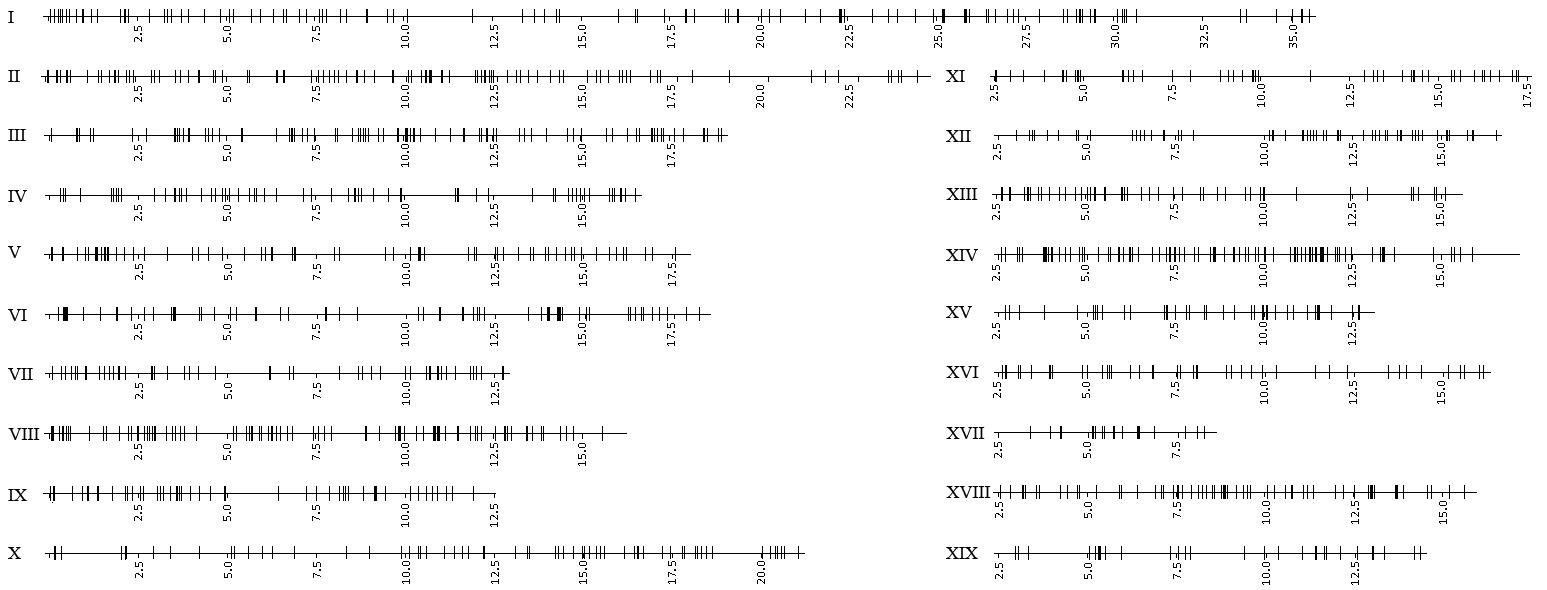
**Representation of the mapping of *C. clementina *BESs hits on poplar chromosomes**. Horizontal lines represent poplar chromosomes in a Mb scale and chromosome numbers are shown on the left. Vertical lines indicate the position of the *C. clementina *BES hits mapped on the poplar genome with BLASTN.

The 1567 BESs that produced significant hits with poplar were mapped on the chromosomes of this species. The representation drawn in Figure 6 showed that citrus sequences were rather uniformly widespread on the 19 poplar chromosomes, an observation that can also be deduced from data in Table 6 that in addition shows that the average number of tags per chromosome was 83 while the distance between tags was 213 kb. Considering that both species have similar genome size, the uniform distribution of the Clementine tags on the poplar chromosomes may suggest that the citrus genome is conveniently represented in the BAC clone set.

**Table 6:** Mapping of citrus BESs hits on poplar chromosomes

Chromosome	Total Length (bp)	n° BES mapped	n° BES/Mb	Average distance between BES
I	35,571,569	136	3.8	283.6
II	24,482,572	138	5.6	192.7
III	19,129,466	116	6.1	178.4
IV	16,625,654	71	4.3	245.3
V	17,991,592	78	4.3	239.9
VI	18,519,121	83	4.5	239.6
VII	12,805,987	63	4.9	208.3
VIII	16,228,216	105	6.5	152.3
IX	12,525,049	66	5.3	186.1
X	21,101,489	80	3.8	282.4
XI	15,120,528	111	7.3	152.1
XII	14,142,880	64	4.5	222.2
XIII	13,101,108	83	6.3	162.4
XIV	14,699,529	112	7.6	134.2
XV	10,599,685	52	4.9	208.2
XVI	13,661,513	61	4.5	241.8
XVII	6,060,117	24	4.0	241.4
XVIII	13,470,992	75	5.6	186.5
XIX	12,003,701	49	4.1	298.3
Average		82.5	5.2	213.5

Following the approach of Lai et al. [20], we used forward and reverse BES read pairs separated by the approximate length of BAC clone inserts (~120 kb), to analyze the microsynteny between *C. clementina *and Arabidopsis, rice and poplar. To be considered as potentially collinear with the target genome, the citrus mate pairs had to map in the heterologous genome into a region comprised between 10 and 300 kb and be also oriented properly. The analyses of the sequences identified 108 Clementine BAC end pairs that met these criteria in poplar, while no one was found in Arabidopsis or rice. Furthermore, the majority of these BES pairs mapped on the *Populus *genome at a distance similar to the insert size of the Clementine libraries, suggesting the microsynteny between citrus and poplar is higher than between citrus and Arabidopsis. These results are striking since *C. clementina *and *A. thaliana *belong to Sapindales and Brassicales orders (eurosids II clade) that probably split approximately 87 MYA, while *P. trichocarpa *belongs to Malpighiales order, (eurosids I clade) that diverged from eurosids II around 109 MYA [64]. Moreover, similar results were obtained by Lai et al [20] with papaya, that also exhibited higher level of colinearity with the poplar than with the Arabidopsis genome despite that *C. papaya *is a basal member of the Brassicales. Although a definitive explanation has not been provided yet, it is currently believed that the genome of *A. thaliana *has undergone a recent whole genome duplication, followed by subsequent gene losses and extensive local gene duplications [18], which might be responsible of the lack of colinearity with other eurosid II species. Comparative genomics with the recently sequenced genome of the grapevine, provides additional evidence that the genome of Arabidopsis has been thoroughly rearranged as related to an ancient angiosperm genome [42]. The fact that papaya, Clementine, grapevine and poplar are long lived, clonally propagated, woody plants, might apparently cause a deceleration of their molecular clocks, resulting in genomes with higher resemblance to the ancestral eurosids genome [17].

## Conclusion

We report here the construction of three genomic BAC libraries of *Citrus clementina*, with three restriction enzymes (*Eco*RI, *Mbo*I and *Hind*III). The number of picked BAC clones (56,000) and the average length of the inserts provide coverage of 19.5 haploid genome equivalents, ensuring a wide representation of the genome of this species. These libraries are adequate for the construction of the physical map of *C. clementina*. The analysis of 28.1 Mb of genomic sequence produced by BAC end sequencing has provided a first insight of the genome organization of *C. clementina*. The repetitive fraction of this species corresponding with transposable elements comprised 12.5% of the genome, while the gene number was estimated to be 31,000. This work also describes a set of 3,814 SSRs and a collection of the first 6,617 putative SNPs described in citrus that may be very useful for positional cloning, genetic and comparative mapping, nucleotide diversity analysis, and association studies. Finally, comparative genomics through gene homology searches has shown that, in spite of their taxonomic classification, microsynteny between *Citrus *and *Populus *is higher than with *Arabidopsis *that is a phylogenetically closer species.

## Methods

### Clementine genotype

*Citrus clementina *(Clementine mandarin, var. clemenules) developing leaves were used for BAC library construction.

### BAC library construction

BAC libraries were constructed from high molecular weight (HMW) genomic DNA processed at Amplicon Express, (Pullman, Washington) using the method described in [65]. DNA digestion was performed with varying amounts of *Mbo*I, *Eco*RI, and *Hind*III to identify appropriate partial digestion conditions. pECBAC1 and contained two FRT and one oriV elements, thus resulting in the pBAC(FRT-oriV) vector [66]. Ligations were transformed into DH10B *Escherichia coli *cells (Invitrogen) and plated on LB agar with chloramphenicol (30 μg/ml), X-gal (20 mg/ml) and IPTG (0.1 M). Clones were robotically picked with a Genomic Solution G3 into 384 well plates containing LB freezing media. Plates were incubated for 18 h, replicated and then frozen at -80°C. The replicated copy was used for BAC end sequencing.

### Insert size estimation

To estimate insert sizes, 10 μl aliquots of BAC miniprep DNA were digested with 5 U of *Not*I enzyme for three h at 37°C. The digestion products were separated by pulsed-field Weld gel electrophoresis (CHEF-DRIII system, Bio-Rad) in a 1% agarose gel in TBE buffer 0,5×. Insert sizes were compared to those of the Lambda Ladder PFG Marker (New England Biolabs). Electrophoresis was carried out for 18 h at 14°C with an initial switch time of 5 s, a Wnal switch time of 15 s, in a voltage gradient of 6 V/cm.

### BAC End Sequencing

BAC clones were inoculated into 96-deep well macroplates and grown for 20 hs at 37°C. Cells were harvested by centrifugation and BACs were purified in 96-well plates by a standard alkaline lysis protocol developed by Genoscope (Paris, France). BAC DNA was precipitated with isopropanol and washed with 70% ethanol. Sequencing was carried out on ABI3730 equipment with "Dye Terminator" process using ABI kit version 3.1. in the Genoscope facility.

### Bioinformatics

The software phred [67]was used for base calling, and Crossmatch for vector masking. Repetitive DNA was identified with the RepeatMasker software [68], using the viridiplantae section of the RepBase Update [69] as database. Assembly was performed with CAP3 [56], using read quality and default parameters. Similarity searches were performed with the standalone version of BLAST [51,70], against the NCBI non redundant protein, nucleotide and EST databases available on November 2007 [71]. Parsing of the BLAST results was performed with the Bio::SearchIO module from the Bioperl package [72]. Coding sequences were annotated with GO terms using Blast2GO [48]. SPUTNIK [50] was used to identify simple sequence repeats (SSRs), and POLYBAYES [62] to search for SNPs.

### SNP validation

DNA extraction was done from leaf tissues of *C. clementina *cv Nules using the DNeasy^® ^Plant Mini Kit (Qiagen).

PCR amplifications of the samples were performed using a Mastercycler epgradiend S thermocycler (Eppendorf) in 100 μL final volume containing 0.025 U/μL of Pfu DNA polymerase (Fermentas), 0.2 ng/μL of genomic DNA, 0.2 mM of each dNTP, 2 mM MgSO4, 75 mM Tris-HCl (pH 8.8), 20 mM (NH4)2SO4, 0.2 μM of each primers. The following PCR program was applied: denaturation at 94° C for 5 min and 35 repeats of the following cycle: 30 s at 94°C, 1 min at 55°C or 60°C (according to primers Tm), 45 s at 72°C; and final elongation step of 4 min at 72°C.

PCR product purification was done directly or after cutting single bands on agarose gel, using respectively QIAquick^® ^PCR Purification Kit and QIAquick^® ^Gel Extraction Kit (Qiagen)

Sequencing was carried out on ABI3730 equipment with "Dye Terminator" process using ABI kit version 3.1. SNPs were identified as double peaks by manual inspection of chromatograms, and subsequently validated

## Authors' contributions

JT was involved in BAC library construction and BAC end sequencing, performed the bioinformatic analyses and drafted the manuscript. MAN performed BAC clone handling and was involved in BAC end sequencing. PO carried out SNP validation. MT coordinated the project and drafted the manuscript.

## Supplementary Material

Additional File 1 Comparative analysis of the 3 BAC libraries. GC content and number of BESs carrying repetitive elements or coding regions are shown for each one of the libraries constructed. Click here for file

Additional file 2 GO Annotations of the coding regions found in BESs. The table shows the GO terms associated with the coding regions identified on the BESs, annotated with B2GO. The description of the GO term, as well as the number sequences associated with each term are shown. Click here for file

Additional File 3 SNP Validation summary. The table shows the details concerning the 24 experimentally validated SNPs, indicating the SNP name (SNP_ID), the contig name (CONTIG) and length (CONTIG LENGTH), the consensus contig sequence (CONT SEQUENCE), the position of the SNP in the consensus sequence (SNP POSITION), the probability of the predicted SNP (P_SNP), the alleles found (ALLELE 1, ALLELE 2), and the primers used for genomic DNA amplification (/FORWARD PRIMER, REVERSE PRIMER). Click here for file

## References

[B1] Nicolosi E, Deng ZN, Gentile A, La Malfa S, Continella G, Tribulato E (2000). Citrus phylogeny and genetic origin of important species as investigated by molecular markers. Theor Appl Genet.

[B2] Gmitter FG (1995). Origin, evolution and breeding of the grapefruit. Plant Breeding Reviews.

[B3] Talon M, Gmitter FG (2008). Citrus Genomics. Int J Plant Genomics.

[B4] Terol J, Conesa A, Colmenero JM, Cercos M, Tadeo F, Agusti J (2007). Analysis of 13000 unique Citrus clusters associated with fruit quality, production and salinity tolerance. BMC Genomics.

[B5] Forment J, Gadea J, Huerta L, Abizanda L, Agusti J, Alamar S (2005). Development of a citrus genome-wide EST collection and cDNA microarray as resources for genomic studies. Plant Mol Biol.

[B6] Chen C, Zhou P, Choi YA, Huang S, Gmitter FG (2006). Mining and characterizing microsatellites from citrus ESTs. Theor Appl Genet.

[B7] Barkley NA, Roose ML, Krueger RR, Federici CT (2006). Assessing genetic diversity and population structure in a citrus germplasm collection utilizing simple sequence repeat markers (SSRs). Theor Appl Genet.

[B8] Jiang D, Zhong GY, Hong QB (2006). Analysis of microsatellites in citrus unigenes. Yi Chuan Xue Bao.

[B9] Ruiz C, Asins MJ (2003). Comparison between Poncirus and Citrus genetic linkage maps. Theor Appl Genet.

[B10] Deng Z, Tao Q, Chang L, Huang S, Ling P, Yu C (2001). Construction of a bacterial artificial chromosome (BAC) library for citrus and identification of BAC contigs containing resistance gene candidates. Theor Appl Genet.

[B11] Yang ZN, Ye XR, Choi S, Molina J, Moonan F, Wing RA (2001). Construction of a 1.2-Mb contig including the citrus tristeza virus resistance gene locus using a bacterial artificial chromosome library of Poncirus trifoliata (L.) Raf. Genome.

[B12] Fagoaga C, Tadeo FR, Iglesias DJ, Huerta L, Lliso I, Vidal AM (2007). Engineering of gibberellin levels in citrus by sense and antisense overexpression of a GA 20-oxidase gene modifies plant architecture. J Exp Bot.

[B13] Katz E, Fon M, Lee YJ, Phinney BS, Sadka A, Blumwald E (2007). The citrus fruit proteome: insights into citrus fruit metabolism. Planta.

[B14] Lliso I, Tadeo FR, Phinney BS, Wilkerson CG, Talon M (2007). Protein changes in the albedo of citrus fruits on postharvesting storage. J Agric Food Chem.

[B15] Tadeo F, Cercos M, Colmenero-Flores JM, Iglesias DJ, Naranjo MA, Rios G (2008). Molecular physiology of development and quality of citrus. Advances in Botanical Research.

[B16] Town CD, Meksem K, Kahl G (2005). Large-scale DNA sequencing. The handbook of Plant Genome Mapping.

[B17] Tuskan GA, DiFazio S, Jansson S, Bohlmann J, Grigoriev I, Hellsten U (2006). The Genome of Black Cottonwood, Populus trichocarpa (Torr. & Gray). Science.

[B18] The Arabidopsis Genome Initiative A (2000). Analysis of the genome sequence of the flowering plant Arabidopsis thaliana. Nature.

[B19] International Rice Genome Sequencing Project (2005). The map-based sequence of the rice genome. Nature.

[B20] Lai C, Yu Q, Hou S, Skelton R, Jones M, Lewis K (2006). Analysis of papaya BAC end sequences reveals first insights into the organization of a fruit tree genome. Mol Genet Genomics.

[B21] Cheung F, Town CD (2007). A BAC end view of the Musa Accuminata genome. BMC Plant Biol.

[B22] Hong CP, Plaha P, Koo DH, Yang TJ, Choi SR, Lee YK (2006). A Survey of the Brassica rapa Genome by BAC-End Sequence Analysis and Comparison with Arabidopsis thaliana. Mol Cells.

[B23] Frelichowski JE, Palmer MB, Main D, Tomkins JP, Cantrell RG, Stelly DM (2006). Cotton genome mapping with new microsatellites from Acala 'Maxxa' BAC-ends. Mol Genet Genomics.

[B24] Marek LF, Mudge J, Darnielle L, Grant D, Hanson N, Paz M (2001). Soybean genomic survey: BAC-end sequences near RFLP and SSR markers. Genome.

[B25] Shultz JL, Samreen K, Rabia B, Jawaad AA, Lightfoot DA (2007). The development of BAC-end sequence-based microsatellite markers and placement in the physical and genetic maps of soybean. Theor Appl Genet.

[B26] Coyne CJ, McClendon MT, Walling JG, Timmerman-Vaughan GM, Murray S, Meksem K (2007). Construction and characterization of two bacterial artificial chromosome libraries of pea (Pisum sativum L.) for the isolation of economically important genes. Genome.

[B27] Nam W, Penmetsa RV, Endre G, Uribe P, Kim D, Cook DR (1999). Construction of a bacterial artificial chromosome library of Medicago truncatula and identification of clones containing ethylene-response genes. Theor Appl Genet.

[B28] Liang H, Fang E, Tomkins J, Luo M, Kudrna D, Kim H (2007). Development of a BAC library for yellow-poplar (Liriodendron tulipifera) and the identification of genes associated with flower development and lignin biosynthesis. Tree Genetics & Genomes.

[B29] Han Y, Gasic K, Marron B, Beever JE, Korban SS (2007). A BAC-based physical map of the apple genome. Genomics.

[B30] Wu C, Sun S, Nimmakayala P, Santos FA, Meksem K, Springman R (2004). A BAC- and BIBAC-Based Physical Map of the Soybean Genome. Genome Res 2004 Feb;14(2):319-26.

[B31] Yim YS, Davis GL, Duru NA, Musket TA, Linton EW, Messing JW (2002). Characterization of Three Maize Bacterial Artificial Chromosome Libraries toward Anchoring of the Physical Map to the Genetic Map Using High-Density Bacterial Artificial Chromosome Filter Hybridization. Plant Physiol.

[B32] Cenci A, Chantret N, Kong X, Gu Y, Anderson OD, Fahima T (2003). Construction and characterization of a half million clone BAC library of durum wheat (Triticum turgidum ssp. durum). Theor Appl Genet.

[B33] Cercos M, Soler G, Iglesias D, Gadea J, Forment J, Talon M (2006). Global Analysis of Gene Expression During Development and Ripening of Citrus Fruit Flesh. A Proposed Mechanism for Citric Acid Utilization. Plant Mol Biol.

[B34] Alos E, Cercos M, Rodrigo MJ, Zacarias L, Talon M (2006). Regulation of color break in citrus fruits. Changes in pigment profiling and gene expression induced by gibberellins and nitrate, two ripening retardants. J Agric Food Chem.

[B35] Sun S, Xu Z, Wu C, Ding K, Zhang HB (2003). Genome properties and their influences on library construction and physical mapping. Proceedings of Plant and Animal Genome XI Conference.

[B36] Ren C, Xu Z, Sun S, Lee MK, Wu C, Scheuring C, Meksem K, Kahl G (2005). Genomic DNA Libraries and Physical Mapping. The handbook of plant genome mapping.

[B37] Chang YL, Tao Q, Scheuring C, Ding K, Meksem K, Zhang HB (2001). An integrated map of Arabidopsis thaliana for functional analysis of its genome sequence. Genetics.

[B38] Chen M, Presting G, Barbazuk WB, Goicoechea JL, Blackmon B, Fang G (2002). An integrated physical and genetic map of the rice genome. Plant Cell.

[B39] Xu Z, Sun S, Covaleda L, Ding K, Zhang A, Wu C (2004). Genome physical mapping with large-insert bacterial clones by fingerprint analysis: methodologies, source clone genome coverage, and contig map quality. Genomics.

[B40] Bausher M, Singh N, Lee SB, Jansen R, Daniell H (2006). The complete chloroplast genome sequence of Citrus sinensis (L.) Osbeck var 'Ridge Pineapple': organization and phylogenetic relationships to other angiosperms. BMC Plant Biology.

[B41] Unseld M, Marienfeld JR, Brandt P, Brennicke A (1997). The mitochondrial genome of Arabidopsis thaliana contains 57 genes in 366,924 nucleotides. Nat Genet.

[B42] Jaillon O, Aury JM, Noel B, Policriti A, Clepet C, Casagrande A (2007). The grapevine genome sequence suggests ancestral hexaploidization in major angiosperm phyla. Nature.

[B43] Rico-Cabanas L, Martinez-Izquierdo JA (2007). CIRE1, a novel transcriptionally active Ty1-copia retrotransposon from Citrus sinensis. Mol Genet Genomics.

[B44] Bernet GP, Asins MJ (2003). Identification and genomic distribution of gypsy like retrotransposons in Citrus and Poncirus. Theor Appl Genet.

[B45] Yuan Q, Ouyang S, Liu J, Suh B, Cheung F, Sultana R (2003). The TIGR rice genome annotation resource: annotating the rice genome and creating resources for plant biologists. Nucl Acids Res.

[B46] Bogunic F, Muratovic E, Brown SC, Siljak-Yakovlev S (2003). Genome size and base composition of five Pinus species from the Balkan region. Plant Cell Reports.

[B47] Carels N, Hatey P, Jabbari K, Bernardi G (1998). Compositional Properties of Homologous Coding Sequences from Plants. J Mol Evol.

[B48] Conesa A, Gotz S, Garcia-Gomez JM, Terol J, Talon M, Robles M (2005). Blast2GO: a universal tool for annotation, visualization and analysis in functional genomics research. Bioinformatics.

[B49] Clement D, Lanaud C, Sabau X, Fouet O, Cunff LL, Ruiz E (2004). Creation of BAC genomic resources for cocoa (Theobroma cacao L.) for physical mapping of RGA containing BAC clones. Theor Appl Genet.

[B50] Abajian C (1994). SPUTNIK. Computer Program.

[B51] Chen C, Zhou P, Choi YA, Huang S, Gmitter FG (2006). Mining and characterizing microsatellites from citrus ESTs. Theor Appl Genet.

[B52] Kijas JMH, Thomas MR, Fowler JCS, Roose ML (1997). Integration of trinucleotide microsatellites into a linkage map of Citrus. Theor Appl Genet.

[B53] Varshney RK, Grosse I, H+ U, Siefken R, Prasad M, Stein N (2006). Genetic mapping and BAC assignment of EST-derived SSR markers shows non-uniform distribution of genes in the barley genome. Theor Appl Genet.

[B54] Mun JH, Kim DJ, Choi HK, Gish J, Debelle F, Mudge J (2006). Distribution of microsatellites in the genome of Medicago truncatula: a resource of genetic markers that integrate genetic and physical maps. Genetics.

[B55] Feingold S, Lloyd J, Norero N, Bonierbale M, Lorenzen J (2005). Mapping and characterization of new EST-derived microsatellites for potato (Solanum tuberosum L.). Theor Appl Genet.

[B56] Huang X, Madan A (1999). CAP3: A DNA sequence assembly program. Genome Res.

[B57] Velasco R, Zharkikh A, Troggio M, Cartwright DA, Cestaro A, Pruss D (2007). A High Quality Draft Consensus Sequence of the Genome of a Heterozygous Grapevine Variety. PLoS ONE.

[B58] Rafalski A (2002). Applications of single nucleotide polymorphisms in crop genetics. Curr Opin Plant Biol.

[B59] Weil MM, Pershad R, Wang R, Zhao S (2004). Use of BAC end sequences for SNP discovery. Methods Mol Biol.

[B60] Abe K, Noguchi H, Tagawa K, Yuzuriha M, Toyoda A, Kojima T (2004). Contribution of Asian mouse subspecies Mus musculus molossinus to genomic constitution of strain C57BL/6J, as defined by BAC-end sequence-SNP analysis. Genome Res.

[B61] Yamamoto K, Narukawa J, Kadono-Okuda K, Nohata J, Sasanuma M, Suetsugu Y (2006). Construction of a Single Nucleotide Polymorphism Linkage Map for the Silkworm, Bombyx mori, Based on Bacterial Artificial Chromosome End Sequences. Genetics.

[B62] Marth GT, Korf I, Yandell MD, Yeh RT, Gu Z, Zakeri H (1999). A general approach to single-nucleotide polymorphism discovery. Nat Genet.

[B63] Pavy N, Parsons LS, Paule C, MacKay J, Bousquet J (2006). Automated SNP detection from a large collection of white spruce expressed sequences: contributing factors and approaches for the categorization of SNPs. BMC Genomics.

[B64] Wikström N, Savolainen V, Chase MW (2001). Evolution of the angiosperms: calibrating the family tree. Proceedings of the Royal Society B: Biological Sciences.

[B65] Tao Q, Wang A, Zhang HB (2002). One large-insert plant-transformation-competent BIBAC library and three BAC libraries of Japonica rice for genome research in rice and other grasses. Theor Appl Genet.

[B66] Frijters CJ, Zhang Z, Damme Mv, Wang L, Ronald PC, Michelmore RW (1997). Construction of a bacterial artificial chromosome library containing large Eco RI and Hin dIII genomic fragments of lettuce. Theor Appl Genet.

[B67] Ewing B, Green P (1998). Base-Calling of Automated Sequencer Traces Using Phred. II Error Probabilities. Genome Res.

[B68] Smit AFA, Hubley R, Green P (1996). RepeatMasker Open-3.0. Computer Program.

[B69] Jurka J, Kapitonov VV, Pavlicek A, Klonowski P, Kohany O, Walichiewicz J (2005). Repbase Update, a database of eukaryotic repetitive elements. Cytogenet Genome Res.

[B70] Altschul SF, Gish W, Miller W, Myers EW, Lipman DJ (1990). Basic local alignment search tool. J Mol Biol.

[B71] National Center for Biotechnology Information. Electronic Citation.

[B72] Stajich JE, Block D, Boulez K, Brenner SE, Chervitz SA, Dagdigian C (2002). The Bioperl toolkit: Perl modules for the life sciences. Genome Res.

