# Supplementary material for: Development of genomic resources for Citrus clementina: Characterization of three deep-coverage BAC libraries and analysis of 46,000 BAC end sequences
Source: BMC Genomics. 2008 Sep 18;9:423. doi: 10.1186/1471-2164-9-423 (PMC2561056; doi:10.1186/1471-2164-9-423)
Supplement: Additional File 1 — Comparative analysis of the 3 BAC libraries. GC content and number of BESs carrying repetitive elements or coding regions are shown for each one of the libraries constructed. [file 1471-2164-9-423-S1.doc]

|  | GC content | BESs with TEs | % BESs with TEs | BESs with BX Hits | BESs with EST Hits | BESs with EST+BX hits | Total coding BESs | % coding BESs |
| --- | --- | --- | --- | --- | --- | --- | --- | --- |
| CCL1 | 35,8% | 3383 | 37% | 2124 | 2144 | 2543 | 6811 | 54% |
| CCER1 | 35,4% | 2589 | 28% | 1796 | 1787 | 2354 | 5937 | 54% |
| CCH3 | 35,9% | 3200 | 35% | 2242 | 2224 | 2971 | 7437 | 59% |
